# Total Glucosides of Paeony Alleviates Acute Kidney Injury by Inhibiting Dendritic Cells and T‐Cell Communication

**DOI:** 10.1155/adpp/8877828

**Published:** 2026-09-30

**Authors:** Tingting Liu, Wei Liang, Yan Zhang, Xiaoqian Wu, Hongwei Zhang, Dongchun Zhu, Yukun Ma, Li Cao, Xiaohua Wang

**Affiliations:** ^1^ Department of Pharmacy The First Affiliated Hospital of Anhui Medical University The Grade 3 Pharmaceutical Chemistry Laboratory of State Administration of Traditional Chinese Medicine Hefei China ahmu.edu.cn; ^2^ School of Pharmaceutical Sciences Anhui Medical University Hefei China ahmu.edu.cn; ^3^ Key Laboratory of Xin’an Medicine Ministry of Education Anhui University of Chinese Medicine Hefei China ahtcm.edu.cn; ^4^ School of Life Sciences State Key Laboratory of Agrobiotechnology The Chinese University of Hong Kong Hong Kong China cuhk.edu.hk; ^5^ Department of Pharmacy Shanghai Hudong Hospital Shanghai China; ^6^ Department of Pharmacy The Fifth Affiliated Hospital of Anhui Medical University Hefei China; ^7^ Department of Ultrasound The First Affiliated Hospital of Anhui University of Chinese Medicine Hefei China ahtcm.edu.cn; ^8^ Department of Pharmacy The 902nd Hospital of the PLA Joint Logistics Support Force Bengbu China

**Keywords:** acute kidney injury, dendritic cells, T cells, total glucosides of paeony

## Abstract

**Aim:**

This study was designed to evaluate the renoprotective action of total glucosides of paeony (TGP) against cisplatin (Cis)‐induced AKI and to delineate the molecular mechanisms.

**Methods:**

AKI was induced in mice via a single intraperitoneal injection of Cis (20 mg/kg). Renal morphological changes were examined using hematoxylin and eosin (H&E) staining, while kidney function and oxidative stress markers were quantitatively assessed. Flow cytometry (FCM) was employed to profile immune cell differentiation and functional status. The mRNA and protein levels of kidney injury–related biomarkers were measured by quantitative real‐time PCR (qPCR) and Western blotting, respectively. To unravel the mechanistic basis, key signaling cascades were probed in both renal tissues and isolated immune cells.

**Results:**

Administration of TGP attenuated renal histological damage and diminished inflammatory cell infiltration in AKI mice. Notably, TGP recalibrated the function of bone marrow–derived dendritic cells (BMDCs), steering them away from a fully mature phenotype. This was evidenced by a marked reduction in the migratory marker C‐C motif chemokine receptor 7 (CCR7) and a reciprocal elevation of the immunoinhibitory checkpoint programmed cell death ligand 1 (PD‐L1). Importantly, this phenotypic shift was linked to TGP‐induced metabolic reprogramming, as DCs from TGP‐treated animals exhibited diminished mitochondrial membrane potential (MMP). The TGP‐mediated DC reprogramming translated into a fundamentally rebalanced T‐cell compartment: The cytotoxic CD8^+^ effector T cell was actively restrained, while the immunosuppressive regulatory T‐cell (T_reg_) subset was preferentially expanded. This modulation was further reinforced by elevated programmed cell death protein 1 (PD‐1) surface expression on T cells. Mechanistic interrogation revealed that TGP concurrently dismantled two interconnected pro‐inflammatory signaling nodes essential for DC‐T cell communication. By attenuating TLR4/MyD88 recruitment, TGP curtailed the downstream nuclear translocation of NF‐κB, thereby limiting the transcriptional output of DC‐derived inflammatory cytokines that drive T‐cell polarization. Concomitantly, TGP directly interfered with the JAK2/STAT3 module, not only diminishing its phosphorylation but also de‐repressing the negative feedback regulator SOCS3. This dual blockade synergistically extinguished the inflammatory amplification loop instigated by Cis in the renal microenvironment.

**Conclusion:**

Collectively, these findings indicate that TGP exerts a protective effect against Cis‐induced AKI by rebalancing immune cell homeostasis and interfering with pro‐inflammatory signaling networks, positioning it as a promising candidate for future AKI pharmacotherapy.

## 1. Introduction

Acute kidney injury (AKI) represents a precipitous deterioration of excretory function, often precipitated by nephrotoxic insults, such as cisplatin (Cis). Beyond the tubular epithelial damage, emerging evidence implicates a dysregulated immune microenvironment as a critical driver of injury propagation [1]. There are about 13 million AKI patients every year in the world in recent years, and the incidence rate is still rising [2]. Previous research shows that the incidence rate of AKI among adults in China is about 11.6%. The high rate of missed diagnosis and lack of treatment has become a huge problem [3].

Despite its potent antitumor activity, Cis’s clinical utility is frequently compromised by dose‐limiting nephrotoxicity, affecting nearly one‐third of treated patients [4]. The ensuing renal injury is not merely a by‐product of direct tubular cytotoxicity; rather, it is profoundly exacerbated by a secondary inflammatory wave, wherein infiltrating immune cells—particularly dendritic cells (DCs) and T lymphocytes—amplify tissue destruction through reciprocal activation loops [5]. Damaged kidney tissue also secretes chemokines that aggravate the infiltration of neutrophils, monocytes, DCs, T cells, etc. [6, 7].

Total glucosides of paeony (TGP), a standardized bioactive extract from *Paeonia lactiflora*, possesses well‐characterized immunomodulatory properties that have been clinically leveraged in autoimmune conditions including rheumatoid arthritis (RA) [8]. Previous studies have focused on the anti‐inflammatory effects of TGP [9]. It has been reported that paeoniflorin and its active ingredients ameliorate kidney injury in collagen‐induced arthritis rats and diabetic kidney disease [10, 11]. Our preliminary experiment demonstrated that TGP modulated the maturation and activation of DCs in vitro. Therefore, investigating the therapeutic effects of TGP in AKI in regulating immune cells is meaningful. These observations led us to hypothesize that TGP mitigates Cis‐induced AKI by intercepting the inflammatory dialog between DCs and T cells, specifically, by restraining DC maturation and curtailing their capacity to prime pro‐inflammatory T‐cell responses, thereby breaking the vicious cycle of immune‐mediated renal injury.

## 2. Materials and Methods

### 2.1. Establishment of the Cis‐Induced AKI Mouse Model

Eight‐week‐old male C57BL/6 mice were housed under specific pathogen‐free conditions at the Laboratory Animal Center of Anhui Medical University. AKI was induced by a single intraperitoneal injection of Cis (20 mg/kg), with TGP or vehicle administered daily via gavage for 7 consecutive days according to a preventive dosing schedule. The experimental groups (*n* = 6 each) comprised vehicle control, Cis model, TGP 80, TGP 160, TGP 320 mg/kg, valsartan, and a valsartan (20 mg/kg) positive control. All procedures were approved by the Animal Experiment Ethics Committee of Anhui Medical University (No. LLSC20190534). All mice were euthanized 8 h after the last administration on the sixth day. The thymus, lymph nodes (LN), spleen, femur, tibia, bilateral kidney tissues, and serum were collected for analysis.

### 2.2. Renal Histology Analysis

The kidneys were collected and quickly fixed in paraformaldehyde fixative for 24 h. About 3 μm paraffin sections were prepared according to the conventional paraffin sectioning process. Hematoxylin and eosin (H&E) staining was used to observe the morphological changes of kidney tissue according to the instructions by optical microscope [12].

### 2.3. Determination of the Weight of the Mice and Their Kidneys, Renal Function, Oxidative Stress, and Inflammatory Factors

Before sacrifice, all mice were weighed. Serum concentrations of creatinine (Scr), blood urea nitrogen (BUN), malondialdehyde (MDA), and superoxide dismutase (SOD) were determined using commercial assay kits obtained from Nanjing Jiancheng Bioengineering Institute (China), with catalog numbers C011‐2‐1, C013‐1‐1, A003‐1‐2, and A001‐3‐2, respectively. All procedures were performed in strict accordance with the manufacturer’s protocols. The serum levels of interleukin‐10 (IL‐10) and IL‐12p70 were tested by enzyme‐linked immunosorbent assay (ELISA) kits (Proteintech, China).

### 2.4. Cell Culture and Treatment

Separate mouse spleens to grind and extract T lymphocytes. Isolate and extract the tibia and femur to collect bone marrow–derived dendritic cells (BMDCs). BMDCs were obtained by culturing in a medium containing cytokines GM‐CSF (20 ng/mL) and IL‐4 (10 ng/mL) for 7 days. All cells were cultured in RPMI 1640 medium (Biochannel, China) containing 10% fetal bovine serum (FBS) and maintained at 37°C in an incubator containing 5% CO_2_ [11].

### 2.5. Flow Cytometry (FCM) Analysis

For immunophenotyping, single‐cell suspensions were stained with fluorochrome‐conjugated antibodies (Multi Sciences, China) against surface markers. T‐cell subsets were delineated as CD3^+^CD4^+^, CD3^+^CD8^+^, and CD4^+^CD25^+^Foxp3^+^T_regs_, while DC maturation status was assessed by gating on CD11c^+^ cells and evaluating MHC‐II and CD86. FITC‐CD11c and PE‐C‐C motif chemokine receptor 7 (CCR7) were used to calculate the expression of CCR7 in CD11c^+^cell gates of DCs. Programmed cell death ligand 1 (PD‐L1) in the CD11c^+^ gate was evaluated using FITC‐CD11c and PE‐CD274 in DCs. Programmed cell death protein 1 (PD‐1) was assessed using APC‐CD3 and PE‐CD279 in T cells.

Mitochondrial membrane potential (MMP) was assessed using the JC‐1 probe with the red‐to‐green fluorescence ratio serving as a quantitative indicator of mitochondrial polarization status. In cells with a high MMP, JC‐1 aggregates and emits red fluorescence (590 nm), whereas in cells with a diminished MMP, the dye remains in its monomeric form, producing green fluorescence (525–530 nm). The ratio of red to green fluorescence was calculated to reflect the metabolic activity of DCs and T lymphocytes.

### 2.6. Western Blot Analysis

Total protein was extracted from renal tissue using phosphatase inhibitor RIPA (Beyotime, China) and PMSF (Beyotime, China). The primary antibodies are detailed in Supporting Table S1. The primary and secondary antibodies are as follows: HRP‐conjugated affinity goat antirabbit IgG (H + L) (Proteintech, China) and HRP‐conjugated affinity goat antirabbit IgG (H + N) (Proteintech, China). Protein samples were detected using ECL chemiluminescence assay kit (Millipore, USA) and analyzed using ImageJ software.

### 2.7. Quantitative Real‐Time PCR (qPCR)

Total RNA was extracted from kidney tissue and DCs using TRIzol reagent (Invitrogen, USA). RNA was reverse‐transcribed into cDNA using a reverse transcription system kit (Vazyme, China). qPCR was performed to measure mRNA levels using SYBR Green qPCR Master Mix (Vazyme, China), following the manufacturer’s protocol. The primers are listed in Supporting Table S2. Gene expression is standardized to the expression of β‐actin.

### 2.8. Cell Counting Kit‐8 (CCK8) Assay and Mixed Lymphocyte Reaction (MLR)

Use the CCK‐8 kit (GLPBIO, China) to evaluate the proliferation of T lymphocytes and the antigen presentation effect of different groups of mouse BMDCs on normal mouse T lymphocytes. Specifically, T lymphocytes from different groups of mice, BMDCs from different groups of mice, and normal mouse T lymphocytes were mixed in proportion and kept in an incubator at 37°C, 5% CO_2_, and 95% air for 24 h. The absorbance at 450 nm was detected using a microplate reader.

### 2.9. Statistical Analyses

All data were subjected to statistical analysis using GraphPad Prism 8.0 and were presented as the mean ± standard deviation (SD). For the comparison of measurement data among multiple groups, the Shapiro–Wilk test was first used to conduct the normality test, and the Levene test was used to conduct the homogeneity of variance test. If the data satisfied the normal distribution and the variance was homogeneous, then the one‐way analysis of variance (ANOVA) was used to compare the overall differences among the groups, while the independent‐samples *t*‐test was employed for two‐group comparisons. Each experiment was replicated at least three times, and a *p* < 0.05 was deemed statistically significant.

## 3. Results

### 3.1. TGP Alleviated Cis‐Induced Kidney Injury and Inflammatory Response

Cis challenge elicited marked elevations in Scr and BUN, which were substantially attenuated by TGP in a dose‐responsive fashion. Concurrently, the kidney‐to‐body weight ratio (renal index, RI), an indicator of renal hypertrophy, was significantly reduced in TGP‐treated animals relative to the Cis models. The commonly used clinical therapeutic drug, valsartan (positive control), also reduced Scr and BUN in Cis‐induced mice. The effect of TGP is similar to that of the positive controls (Figure 1A). Compared with the models, TGP treatment alleviated oxidative stress, with a decrease in MDA and an increase in SOD (Figure 1B). We used the semiquantitative scoring system [13] to assess renal tubular damage. H&E staining of renal tissue showed that TGP and valsartan reduced Cis‐induced tubular injury, improved tubular necrosis, tubular dilation, and reduced epithelial cell atrophy (Figure 1C). At the same time, TGP decreased the pro‐inflammatory cytokine IL‐12 and increased the anti‐inflammatory cytokine IL‐10 (Figure 1D). In addition, TGP inhibited the protein level of renal injury marker (KIM‐1), which indicated that TGP could repair kidney damage and alleviate inflammation (Figure 1E). We evaluated the expression of key renal transporters involved in Cis uptake (organic cation transporter 2, OCT2/SLC22A2) and efflux (multidrug and toxin extrusion protein 1, MATE1/SLC47A1). The results showed that Cis downregulated the expression of both transporters. Conversely, TGP treatment significantly upregulated the expression of the efflux transporter MATE1, while partially restoring OCT2 expression. This preferential upregulation of MATE1 indicated that TGP attenuated nephrotoxicity by enhancing the efflux of Cis from renal tubular cells, thereby reducing intracellular Cis accumulation (Figure 1F). The results indicated that TGP exhibited renal protective and anti‐inflammatory effects on Cis‐induced AKI.

**FIGURE 1:**
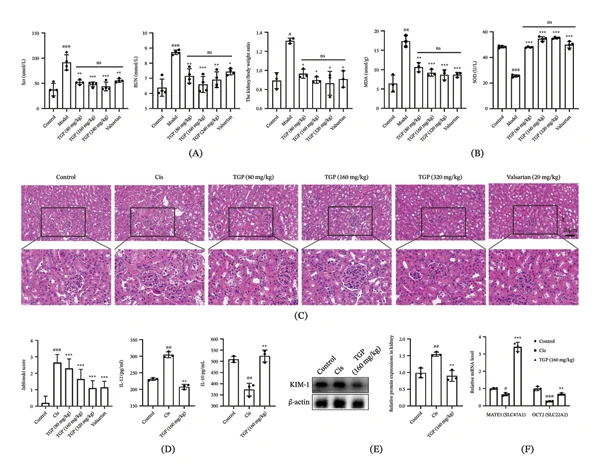
Effect of TGP on Cis‐induced renal injury and inflammatory response. (A) Scr (*n* = 4), BUN (*n* = 4), and kidney index (*n* = 3). (B) Serum levels of MDA (*n* = 3) and SOD (*n* = 4). (C) H&E staining images and histopathology injury scoring (original magnification 200x; scale bar for initial image: 20 μm) (*n* = 3). (D) Serum inflammatory cytokine secretion (*n* = 3). (E) Kidney injury molecule‐1 (KIM‐1) protein expression (*n* = 3). (F) Relative mRNA levels of MATE1 (SLC47A1) and OCT2 (SLC22A2) (*n* = 3). Data are expressed as mean ± SD. ^#^ *p* < 0.05, ^##^ *p* < 0.01, ^###^ *p* < 0.001 compared with the controls; ^∗^ *p* < 0.05, ^∗∗^ *p* < 0.01, ^∗∗∗^ *p* < 0.001 compared with the models; ns, not significant.

### 3.2. TGP Regulated T‐Cell Differentiation and Function in AKI Mice

We quantitatively assessed the effects of TGP on T‐cell subset distribution and differentiation in AKI mice. As shown, Cis challenge led to a marked increase in the proportion of CD8^+^T cells within the spleen and LNs, an effect that was effectively counteracted by TGP administration (Figure 2A). In parallel, TGP significantly downregulated the renal mRNA expression of granzyme B (GzmB), a key cytotoxic effector molecule of CD8^+^T cells (Figure 2B). In contrast, the CD4^+^T‐cell population remained largely unaffected by either treatment (Figure 2A). Regarding T_regs_, Cis exposure reduced their frequencies in the spleen, thymus, and kidney, whereas TGP treatment significantly restored these proportions. Notably, this restorative effect was not observed in the LNs (Figure 2C). Consistent with this immunomodulatory profile, TGP treatment elevated Foxp3 mRNA levels in renal tissue, indicative of enhanced immune tolerance (Figure 2D). Furthermore, TGP markedly suppressed T‐cell proliferative capacity (Figure 2E).

**FIGURE 2:**
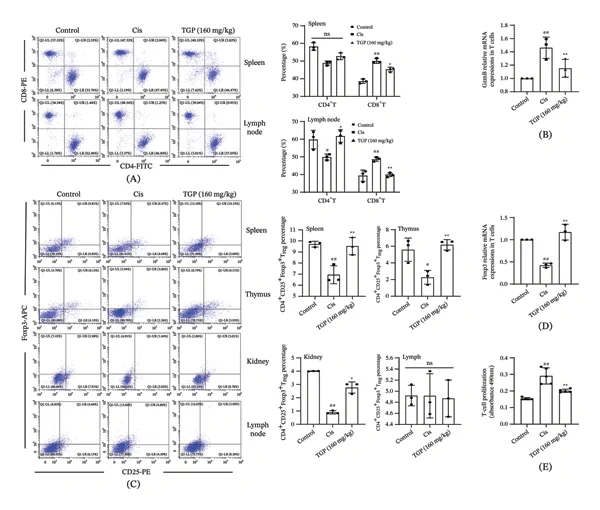
Effects of TGP on T lymphocyte differentiation in Cis‐induced AKI mice. (A) Percentages of CD4^+^T cells and CD8^+^ T cells in the spleen and LN (*n* = 3). (B) Expression of GzmB mRNA in T lymphocytes (*n* = 3). (C) Percentages of T_regs_ in the spleen, thymus, LN, and kidney (*n* = 3). (D) Expression of Foxp3 mRNA in T lymphocytes (*n* = 3). (E) The proliferation of T cells (*n* = 5). Data are expressed as mean ± SD. ^#^ *p* < 0.05, ^##^ *p* < 0.01 compared with the controls; ^∗^ *p* < 0.05, ^∗∗^ *p* < 0.01 compared with the models; ns, not significant.

### 3.3. TGP Regulated the Maturation and Function of DCs in AKI

We next evaluated the effect of TGP on the maturation status of both renal DCs and BMDCs in the AKI model. As presented, Cis challenge significantly upregulated the surface expression of CD86 and MHC‐II on renal DCs. TGP administration effectively attenuated the MHC‐II levels on these cells. In parallel, TGP treatment markedly suppressed the phenotypic maturation of BMDCs relative to the Cis group (Figure 3A). Furthermore, TGP downregulated the chemokine receptor CCR7 on DCs, suggesting a modulatory effect on the migratory capacity of DCs in the context of AKI (Figure 3B). Additionally, TGP treatment elevated the transcript levels of indoleamine‐2,3‐dioxygenase (IDO), a well‐characterized negative immunoregulatory molecule, in BMDCs, which is consistent with a shift toward an immature DC phenotype (Figure 3C).

**FIGURE 3:**
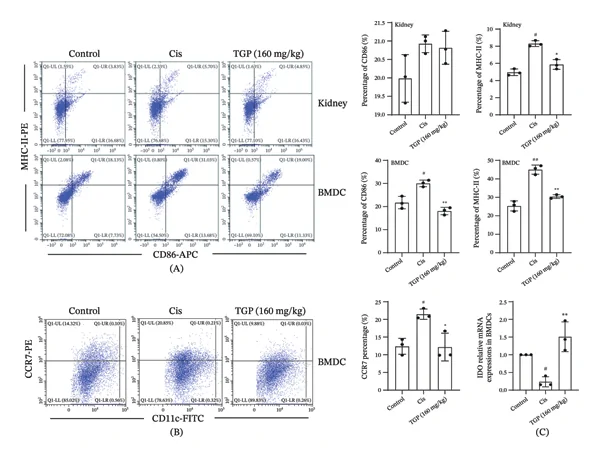
Effects of TGP on DC maturation and differentiation in Cis‐induced AKI mice. (A) Expression of MHC‐II and CD86 on renal DCs and BMDCs. (B) Expression of CCR7 on BMDCs. (C) Expression of IDO mRNA in BMDCs. Data are expressed as mean ± SD (*n* = 3). ^#^ *p* < 0.05, ^##^ *p* < 0.01 compared with the controls; ^∗^ *p* < 0.05, ^∗∗^ *p* < 0.01 compared with the models; ns, not significant.

### 3.4. TGP Downregulated the Communication Between BMDCs and T Cells

As professional antigen‐presenting cells, DCs orchestrate inflammatory immune responses by delivering antigens to T cells. Our data demonstrated that TGP upregulated the surface expression of the negative co‐stimulatory molecule PD‐1 on T cells (Figure 4A), as well as its ligand PD‐L1 on BMDCs (Figure 4B). Consistently, MLR assays revealed that TGP impaired the capacity of DCs to stimulate T‐cell proliferation (Figure 4C). At the transcript level, TGP treatment concurrently elevated the renal mRNA expression of multiple inhibitory co‐stimulatory genes, including PD‐1, PD‐L1, and cytotoxic T‐lymphocyte‐associated antigen‐4 (CTLA‐4) (Figure 4D). To further explore the metabolic underpinnings of these immunomodulatory effects, we employed the JC‐1 probe to evaluate MMP, a reliable indicator of cellular energy status. TGP administration resulted in a marked reduction in MMP in both T cells (Figure 4E) and BMDCs (Figure 4F). Collectively, these findings suggested that TGP‐induced metabolic perturbation impaired the activation and functional competence of both DCs and T cells, thereby ameliorating the inflammatory milieu characteristic of AKI.

**FIGURE 4:**
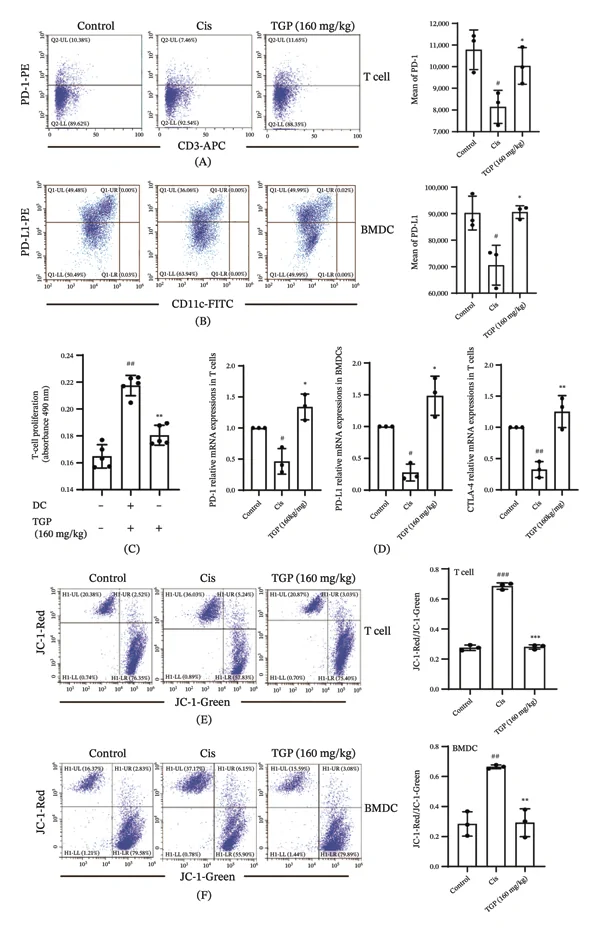
Effects of TGP on the interaction between BMDCs and T cells in Cis‐induced AKI mice. (A) Expression of PD‐1 on splenic T cells (*n* = 3). (B) Expression of PD‐L1 in BMDCs (*n* = 3). (C) MLR assay (*n* = 5). (D) Expression of PD‐1 and CTLA‐4 mRNA in splenic T lymphocytes, and PD‐L1 mRNA in BMDCs (*n* = 3). (E) MMP of T cells (*n* = 3). (F) MMP of DCs (*n* = 3). Data are expressed as mean ± SD. ^#^ *p* < 0.05, ^##^ *p* < 0.01, ^###^ *p* < 0.001 compared with the controls; ^∗^ *p* < 0.05, ^∗∗^ *p* < 0.01, ^∗∗∗^ *p* < 0.001 compared with the models; ns, not significant.

### 3.5. TGP Inhibited the Toll‐Like Receptor 4 (TLR4)‐MyD88‐NF‐κB and JAK2‐STAT3‐SOCS3 Pathways in AKI

The TLR4‐MyD88‐NF‐κB pathway participates in the DC maturation [14]. The JAK2‐STAT3 pathway is associated with inflammation in AKI [15, 16]. The results indicated that TGP inhibited the protein expression of TLR4, MyD88, and NF‐κB in the BMDCs (Figure 5A) and kidney (Figure 5B) in AKI. In addition, after Cis treatment, the protein expression and phosphorylation of JAK2 and STAT3 in the kidney and BMDCs increased with the decrease of the negative regulatory molecule SOCS3. Treatment with TGP reversed the changes and upregulated the expression of SOCS3 (Figure 5C,D). In conclusion, TGP might alleviate inflammation and regulate immunity in AKI by modulating DCs and T‐cell communication through TLR4‐MyD88‐NF‐κB and JAK2‐STAT3‐SOCS3 signals.

**FIGURE 5:**
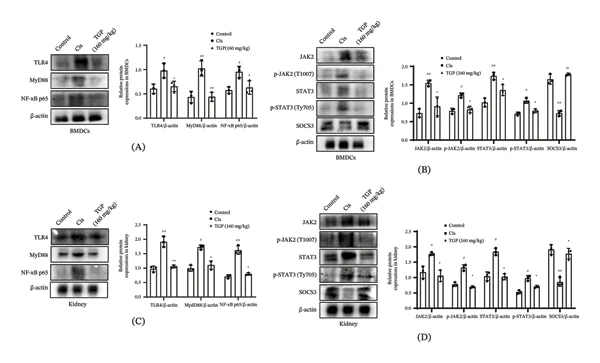
Effects of TGP on the TLR4‐MyD88‐NF‐κB and JAK2‐STAT3‐SOCS3 pathways in Cis‐induced AKI mice. (A) Expression of TLR4, MyD88, and NF‐κB proteins in BMDCs. (B) Expression of JAK2, STAT3, and SOCS3 proteins in BMDCs. (C) Expression of TLR4, MyD88, and NF‐κB proteins in kidney tissues. (D) Expression of JAK2, STAT3, and SOCS3 proteins in kidney tissues. Data are expressed as mean ± SD (*n* = 3). ^#^ *p* < 0.05, ^##^ *p* < 0.01 compared with the controls; ^∗^ *p* < 0.05, ^∗∗^ *p* < 0.01 compared with the models; ns, not significant.

## 4. Discussion

AKI was characterized by a rapid decline in renal function, which posed a life‐threatening risk. Cis, as a broad antitumor drug, accumulated in proximal renal tubular epithelial cells and induced severe nephrotoxicity, which became a leading cause of drug‐induced AKI [17]. Cis increased the production of cytokines and chemokines in the kidney, accompanied by extensive infiltration of inflammatory cells. Subsequently, the recruitment of neutrophils, monocytes, DCs, T cells, and other immune cells occurred [18]. The inflammatory cells in the kidney amplified the immune response by releasing inflammatory mediators, such as TNF‐α, IFN‐γ, IL‐12, and GzmB, which exacerbated renal damage and renal failure [19]. Radix Paeoniae Alba is a commonly used traditional Chinese medicine in clinical practice [20]. The TGP includes paeoniflorin, paeoniflorin glycoside, and paeoniflorin glycoside. TGP exhibits significant anti‐inflammatory and immune regulatory effects in clinical practice [21, 22]. The results of this study showed that in the Cis‐induced AKI animal model, the epithelial cells of the proximal renal tubules in the mice exhibited vacuolar degeneration, nuclear condensation, and tubular formation in the tubular lumen. Scr and BUN levels in the serum and kidney injury marker KIM‐1 protein in the kidney tissue of AKI mice increased significantly. TGP treatment improved renal function, alleviated pathological damage, and reduced the expression of KIM‐1 in the AKI mice, which indicated its protective effect against Cis‐induced AKI.

There are various immune cells involved in AKI, including neutrophils, monocytes, DCs, natural killer cells, CD4^+^T cells, and T_regs_ [8, 23]. Immune cells play different functions according to their characteristics, among which DCs can damage the kidneys by secreting cytokines or activating T cells [24]. T_regs_ have immunosuppressive functions, which protect the kidneys by inhibiting T‐cell function and releasing anti‐inflammatory factors, such as IL‐10 [25]. Improving the excessive inflammatory response in the kidneys by regulating the number and function of immune cells is an important way to improve AKI [26]. The results of this study showed that TGP inhibited the maturation, activation, and migration of BMDCs and upregulated the expression of negative regulatory molecule PD‐L1, which weakened the activation and antigen presentation to T cells. The secretion of pro‐inflammatory factors, such as IL‐12 and IFN‐γ, was reduced, and the levels of anti‐inflammatory factors, such as IL‐10, increased after TGP treatment. This demonstrates the important role of DCs in the renal immune inflammatory response to the effect of TGP on AKI.

TLRs are a type of pattern recognition receptor. Previous studies found that TLR4 plays an important role in Cis‐induced damage to renal tubular epithelial cells [27]. The low expression of TLR4 could significantly alleviate inflammatory cell infiltration in Cis‐induced renal damage through MyD88‐dependent or nondependent signaling pathways that mediate immune responses [28]. The signal dependent on MyD88 mainly activates and induces DCs to secrete pro‐inflammatory cytokines, such as IFN‐γ and IL‐12 [29]. After TLR4 activation, it forms the MD2/TLR4/CD14 complex with MD2 and CD14 molecules, causing MyD88 to aggregate on the MD2/TLR4/CD14 complex. MyD88 then recruits IRAKs to the MD2/TLR4/CD14 complex by interacting with related kinases (IRAKs), where IRAK1 is phosphorylated by IRAK4 and activated [30]. The hyperphosphorylated IRAK1 binds to TRAF6 and activates it, activating downstream pathways and leading to activation of NF‐κB inhibitor kinase 2, resulting in phosphorylation of IκB [31]. Once phosphorylated, IκB is ubiquitinated by a multimeric ubiquitin ligase, degraded by the proteasome, and the nuclear localization sequence of NF‐κBp65 is exposed. At this time, p65 undergoes nuclear translocation, connects to a series of homologous DNA sequences of promoters, and recruits transcription components required to induce gene expression, regulating the transcription levels of inflammatory factors, such as IL‐12, IFN‐γ, and TNF‐α. The increase in the expression levels of these inflammatory factors can further stimulate the level of NF‐κB activation, leading to further amplification of the initial inflammatory signal and promoting the maturation and activation of DCs [32, 33]. Furthermore, the JAK‐STAT signaling cascade represents a principal axis through which the majority of inflammatory cytokines exert their biological actions [34]. The results of this study showed that TGP downregulated the expression of TLR4 protein in the kidneys and DCs, reduced the activation of NF‐κBp65, downregulated the levels of JAK2 and STAT3 proteins and their phosphorylation, upregulated the expression of negative regulatory factor SOCS3 protein, and alleviated kidney injury. TGP may affect regulatory circuits involved in the interaction of these two signals to maintain immune homeostasis during AKI.

The study highlighted the unique advantages of TGP as a multitarget natural compound. Unlike conventional drugs, TGP, only a single inflammatory mediator, exerted an overall regulatory effect by reshaping the DC‐T cell immune axis, thereby restoring immune homeostasis in the renal microenvironment. In addition, such dual regulations of the TLR4/NF‐κB and JAK2/STAT3 pathways indicated that TGP blocked the inflammatory cascade at an upstream level. Given that TGP is already clinically indicated for autoimmune disorders, such as RA, our findings extend its therapeutic repertoire to a novel application. Specifically, TGP warrants consideration as a renoprotective adjunct in chemotherapy regimens, as it can attenuate Cis‐induced nephrotoxicity. This strategy may enhance patients’ quality of life and adherence to therapy.

Collectively, our findings establish TGP as a dual‐action immunomodulator that intercepts Cis‐induced renal injury at the critical juncture of innate‐adaptive immune crosstalk. By simultaneously targeting the TLR4‐driven DC maturation checkpoint and the JAK2‐STAT3 inflammatory rheostat, TGP not only ablates the pathological activation of DCs but also reprograms the downstream T‐cell effector landscape toward a tolerogenic profile. Given the clinical availability of TGP and its established safety profile in autoimmune conditions, our data advocate for its repurposing as a renoprotective adjuvant in Cis‐based chemotherapeutic regimens, warranting further translational investigation (Figure 6).

**FIGURE 6:**
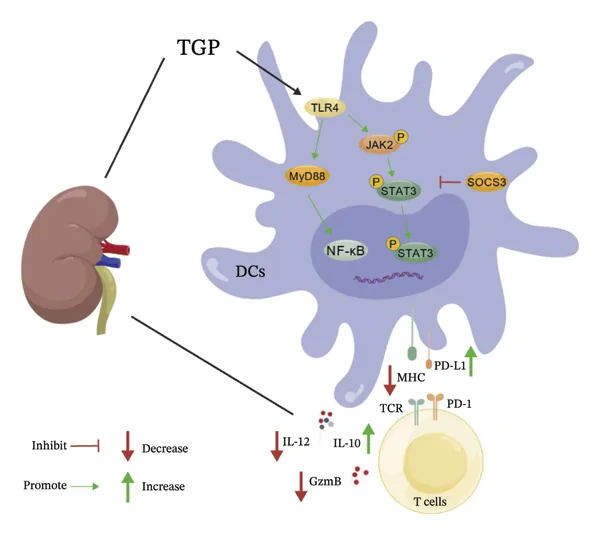
Schematic illustration of TGP treatment in AKI. TGP targeted TLR4‐MyD88‐NF‐κB and JAK2‐STAT3‐SOCO3 signaling in DCs, followed by inhibiting the crosstalk between DCs and T cells to alleviate AKI.

## Author Contributions

Tingting Liu participated in the study design, analyzed the data, involved in material support, drafted the manuscript, participated in the technical guidance and coordination, and supervised the study. Wei Liang carried out the experiments, analyzed the data, and drafted the manuscript. Xiaohua Wang participated in the technical guidance and coordination. Yan Zhang, Xiaoqian Wu, Hongwei Zhang, Dongchun Zhu, Yukun Ma, and Li Cao carried out parts of the experiments.

## Funding

This work was supported by the National Natural Science Foundation of China (No. 81903622), the Nature Science Foundation of Anhui Province of China (No. 2508085MH221), the Anhui Provincial Scientific Research Project on Traditional Chinese Medicine Inheritance and Innovation (No. 2025CCCX026), the Open Fund of Key Laboratory of Xin’an Medicine, Ministry of Education, P.R. China (Anhui University of Chinese Medicine) (No. 2024xayx06), the Anhui Provincial Quality Engineering Education Research Project (No. 2023jyxm0981), the Outstanding Talent Cultivation Program for Universities of Department of Education of Anhui Provincial (No. gxgwfx2021016), and the Bengbu Municipal Health Research Project (No. BBWK2025A04).

## Disclosure

All authors read and approved the final manuscript.

## Conflicts of Interest

The authors declare no conflicts of interest.

## Supporting Information

Additional supporting information can be found online in the Supporting Information section.

## Supporting information


**Supporting Information** Supporting Table 1 and Table 2 may be found at Additional Supporting Information.

## Data Availability

The data that support the findings of this study are available from the corresponding author upon reasonable request.
