# Supplementary material for: Total Glucosides of Paeony Alleviates Acute Kidney Injury by Inhibiting Dendritic Cells and T‐Cell Communication
Source: Adv Pharmacol Pharm Sci. 2026 Sep 30;2026:8877828. doi: 10.1155/adpp/8877828 (PMC13627981; doi:10.1155/adpp/8877828)
Supplement: Supplementary file 1 [file ADPP-2026-8877828-s001.zip › Supplementary Table 1.doc]

**Supplementary Table 1. Antibody information for western blot**

| **Antibody** | **Manufacturer** | **Catalog number** | **Dilution used** |
| --- | --- | --- | --- |
| β-actin | Proteintech | 66009-1-lg | 1:10000 |
| TLR4 | proteintech | 66350-1-Ig | 1:500 |
| MyD88 | Immunoway | Q6Y1S1 | 1:1000 |
| NF-κB | Abmart | T55034 | 1:1000 |
| JAK2 | Immunoway | YT2429 | 1:1000 |
| p-JAK2 | Immunoway | YP0155 | 1:1000 |
| STAT3 | Proteintech | 10253-2-AP | 1:1000 |
| p-STAT3 | Immunoway | YP0251 | 1:1000 |
| SOCS3 | Proteintech | 14025-1-AP | 1:1000 |
| KIM-1 | Abmart | HAVCR1 | 1:1000 |
