# Supplementary material for: Total Glucosides of Paeony Alleviates Acute Kidney Injury by Inhibiting Dendritic Cells and T‐Cell Communication
Source: Adv Pharmacol Pharm Sci. 2026 Sep 30;2026:8877828. doi: 10.1155/adpp/8877828 (PMC13627981; doi:10.1155/adpp/8877828)
Supplement: Supplementary file 1 [file ADPP-2026-8877828-s001.zip › Revised Supplementary Table 2.doc]

**Supplementary Table 2. The sequences of primers**.

| **Gene name** | **Forward sequence** | **Reverse sequence** |
| --- | --- | --- |
| *β-actin* | ACACCTTCTACAATGAGCTG | CTGCTTGCTGATCCACATCT |
| *Foxp3* | GACATCCCATATTCTCCCA | GACGTGAAGCCTAGACAGC |
| *PD-1* | CGCCTGAGTCCCAGCAACCAG | GCTCCAGGGCTCTCCTCGAT |
| *CTLA-4*  *GzmB*  *IDO*  *PD-L1*  *OCT2*  *MATE1* | TGGGCTTCCTAGATTACCCCTT  GACCCTACATGGCCTTACTTTC  TTGCCAAATGTGACTGCT  CAAAGAATTTTGGTTGTGGA  CTTGCTGTCCTTGTCTGCTC  GCTTCCTAGAGGAGCTGCG | AATCTGCGTCCCGTTGCCCAT  TATGAAGCCAGTCTTTGCAGTC  CAAACTAATGACAAATCCCT  AGCTTCTCCTCTCTCTTGGA  AGAGCCTTCCCTTTGGTCTC  GACAAGCCATGTCCCACTGA |
